# Cortical Network Disruption Is Minimal in Early Stages of Psychosis

**DOI:** 10.1093/schizbullopen/sgae010

**Published:** 2024-04-22

**Authors:** Peter C Van Dyken, Michael MacKinley, Ali R Khan, Lena Palaniyappan

**Affiliations:** Neuroscience Graduate Program, Schulich School of Medicine and Dentistry, Western University, London, ON, Canada; Lawson Health Research Institute, London Health Sciences Centre, London, ON, Canada; Robarts Research Institute, Schulich School of Medicine and Dentistry, Western University, London, ON, Canada; Department of Medical Biophysics, Schulich School of Medicine and Dentistry, Western University, London, ON, Canada; Robarts Research Institute, Schulich School of Medicine and Dentistry, Western University, London, ON, Canada; Department of Medical Biophysics, Schulich School of Medicine and Dentistry, Western University, London, ON, Canada; Department of Psychiatry, Douglas Mental Health University Institute, McGill University, London, ON, Canada; Department of Psychiatry, Schulich School of Medicine and Dentistry, Western University, London, ON, Canada

**Keywords:** schizophrenia, diffusion-weighted imaging, structural connectivity, graph theory, first episode psychosis, connectome

## Abstract

**Background and Hypothesis:**

Schizophrenia is associated with white matter disruption and topological reorganization of cortical connectivity but the trajectory of these changes, from the first psychotic episode to established illness, is poorly understood. Current studies in first-episode psychosis (FEP) patients using diffusion magnetic resonance imaging (dMRI) suggest such disruption may be detectable at the onset of psychosis, but specific results vary widely, and few reports have contextualized their findings with direct comparison to young adults with established illness.

**Study Design:**

Diffusion and T1-weighted 7T MR scans were obtained from *N* = 112 individuals (58 with untreated FEP, 17 with established schizophrenia, 37 healthy controls) recruited from London, Ontario. Voxel- and network-based analyses were used to detect changes in diffusion microstructural parameters. Graph theory metrics were used to probe changes in the cortical network hierarchy and to assess the vulnerability of hub regions to disruption. The analysis was replicated with *N* = 111 (57 patients, 54 controls) from the Human Connectome Project-Early Psychosis (HCP-EP) dataset.

**Study Results:**

Widespread microstructural changes were found in people with established illness, but changes in FEP patients were minimal. Unlike the established illness group, no appreciable topological changes in the cortical network were observed in FEP patients. These results were replicated in the early psychosis patients of the HCP-EP datasets, which were indistinguishable from controls in most metrics.

**Conclusions:**

The white matter structural changes observed in established schizophrenia are not a prominent feature in the early stages of this illness.

## Introduction

The neuropathology of schizophrenia comprises a generalized dysconnectivity between brain regions.^1,2^ The current consensus suggests a loss or “subtle randomization” of functional relationships across the brain,^3–7^ predominantly affecting highly connected neural hubs. This causes alterations of the cortical hierarchy,^5,8^ hampering the integrated processing of information and causing disorganization of thoughts, speech, and behavior.^9,10^

White matter (WM) pathology has long been explored as a causative or mediating factor of schizophrenia.^10^ Two decades of diffusion magnetic resonance imaging (dMRI) research, including a mega-analysis of 1963 individuals with schizophrenia,^11^ have established the disruption of WM integrity as a robust feature of established schizophrenia.^2,12–15^ Although the histological implications of these findings are not entirely clear, they may involve some combination of the degradation of myelin sheaths on long axonal projections,^16^ decreased axonal density,^17^ or increased fiber disorganization.^18^

If these changes arise early in schizophrenia, they may reflect causative, pathological processes, and their precise quantification may aid early detection. Such an idea is easily motivated by our current developmental understanding of the disease.^19,20^ A substantial genetic component^21^ and associations with childhood^22^ and perinatal trauma^23^ suggest schizophrenia arises from pathological processes beginning very early in life. Indeed, the first psychotic episode is generally preceded by a subthreshold phase dominated by negative symptoms and poor premorbid functioning that can last for several years.^24^ We thus might expect structural changes to have accumulated by the time of the first psychotic episode, an idea forming the basis of the neurodevelopmental hypothesis.^25^ If so, such changes may be detectable at the time of illness onset.^26^

Previous dMRI studies in first-episode psychosis (FEP) have thus far converged on a report of reduced fractional anisotropy (FA) in FEP,^27^ but the location and scale of these findings vary across studies.^28–39^ Other reports have studied changes in the number of streamlines connecting cortical regions, a metric that gives insight into the anatomical makeup of the structural connectome.^40^ Results from such analyses have been relatively modest compared to FA. Reductions and elevations of streamline counts are observed in scattered connections^41–43^ and the connectivity of cortical hubs is slightly reduced.^44,45^

Common to all the above studies is the relative paucity of findings compared to those observed in chronic patients. In studies analyzing both groups together, disruption is consistently greater in older, more chronic patients compared to those with FEP.^42,46–48^ This concurs with larger cross-sectional studies showing a relation between disease-related microstructural changes and duration of illness.^11,49^ These prior studies, however, have been limited by small FEP samples (*n* ≤ 20), limiting the power to detect differences,^42,47^ or by the inclusion of patients exposed to several months of treatment.^46,48^ Studies of early-onset psychosis (rather than adult-onset psychosis), which include a substantial number of adolescent patients, have been similarly confounded by notable antipsychotic exposure (eg, 90% exposure, >6 months).^50^

The lack of a consistent, anatomically localized disruption in FEP (“lesion-like” changes) may mask a more reproducible topological effect (architectural changes). Individual deficits may be anatomically scattered, reflecting high interindividual variability difficult to observe at the group-level, yet still produce a converging effect on the overall topology.^8,51^ Disruption in chronic patients is already known to be topologically biased, with highly connected hub nodes bearing the greatest burden.^12,52^ Previous work has mostly focused on anatomically localized disruption, and no prior studies of FEP patients have studied both anatomical and topological disruption in concert.

Finally, the use of single-site dMRI datasets hinders attempts at replication, as findings may reflect the unique acquisition parameters of the dataset.^53^ The availability of high quality, open-access datasets allows us to replicate observations to ensure robustness of both positive and negative findings. The recently released Human Connectome Project-Early Psychosis (HCP-EP) dataset represents the first such openly available dataset specifically of individuals shortly after their first psychotic episode (<5 years since diagnosis). Its diffusion data has not yet been analyzed in any major connectivity study of early psychosis (EP).

In this study, we analyzed geometric and topological disruption in the brains of FEP and EP patients using the Tracking Outcomes in Psychosis (TOPSY) dataset, a 7T dMRI dataset of untreated FEP patients (recruited from a rapid care psychosis service and scanned in the week of first contact), age-matched controls, and chronic patients, and the HCP-EP dataset. We hypothesize that WM connectivity of FEP and EP patients will be disrupted relative to healthy counterparts.

## Methods

### Data

#### TOPSY Dataset.

FEP patients, chronic patients, and healthy controls (HCs) were recruited from an established cohort enrolled in the Prevention and Early Intervention Program for Psychoses (PEPP) in London, Ontario. This is a high fidelity, early intervention program that uses an intense case management model, receiving all incident cases of psychosis in the defined catchment area, with first contact made within 48 h of referral. Inclusion criteria for study participation were as follows: for FEP patients: individuals experiencing their first psychotic episode, with no more than 14 days of cumulative lifetime antipsychotic exposure, ability to provide informed consent, no major head injuries (leading to a significant period of unconsciousness or seizures), no known neurological disorders, and no concurrent substance use disorder. Participants were not explicitly instructed to abstain from substances, and patients on non-antipsychotic prescription medication were not excluded. All participants provided written, informed consent prior to participation as per approval provided by the Western University Health Sciences Research Ethics Board, London, Ontario.

For FEP, the mean lifetime total defined daily dose exposure (DDD × days on medication) for antipsychotic use was 1.16 DDD days with 24 patients (47.4%) being completely antipsychotic naive at the time of scanning. Of those who had started antipsychotic treatment, (*N* = 34; 70.2%), the median lifetime exposure in DDD days was 0.93 days (range of 0.4–11.3 DDD days), indicating that in most cases over lifetime, less than 1 day’s worth of effective antipsychotic dose was administered. Patients with established (chronic) schizophrenia consisted of clinically stable patients on long-acting injectable medications with 3 or more years since illness onset, no recorded hospitalization in the past year, and receiving community-based care from physicians affiliated to a first-episode clinic (PEPP, London Ontario). While many studies have focused on chronic schizophrenia patients in their 40s or 50s, our approach enabled us to reduce (even if we cannot fully avoid) the age gap with FEP and HC groups. Patient consensus diagnosis was established using the best estimate procedure described by Leckman et al^54^ and confirmed after 6 months of treatment. The diagnosis of schizophrenia was based on the DSM-5 criteria.

HCs were recruited through posters and word-of-mouth advertising. They had no personal history of mental illness, no current use of medications, and no first-degree relatives with a history of psychotic disorders. HCs were group matched to the FEP cohort for age and parental socioeconomic status (the National Statistics Socioeconomic Classification: 5-class version). Like their FEP counterparts, those with a history of substance use disorders in the past 12 months, significant head injury, or neurological disorders were excluded.

Data were acquired with a head-only, neuro-optimized 7T MRI (Siemens MAGNETOM Plus, Erlangen, Germany) using dMRI and T1-weighted (T1w) imaging protocols. T1w data were collected using an MP2RAGE sequence^55^ at 0.75 mm isotropic resolution, echo time = 2.83 ms, repetition time = 6 s, field of view = 240 × 240 mm, number of slices = 208. The T1w image was reconstructed using the robust algorithm introduced by O’Brien et al^56^ Diffusion data were acquired with an echo-planar imaging (EPI) sequence at 2 mm isotropic resolution, echo time = 50.2 ms, repetition time = 5.1 s, field of view = 208 mm, number of slices = 72, MB acceleration factor = 2, flip angle = 90. 64 directions were acquired in both the AP and PA directions at *b* = 1000, along with 2 *b* = 0 images. Gradient nonlinearity correction was applied to all acquisitions using in-house software.

17 subjects (1 HC, 13 FEP, 3 chronic) failed to complete the acquisition protocol, 1 further FEP subject was excluded due to poor data quality. Four FEP subjects that did not receive a schizophrenia spectrum disorder diagnoses (2 with major depressive disorder, 2 with bipolar) were also excluded from analysis.

#### HCP-EP Dataset.

HCP-EP data were accessed according to the Data Use Certification issued by the NIMH Data Archive. Complete inclusion and exclusion criteria can be found in the dataset manual (retrievable at time of publication at https://humanconnectome.org/study/human-connectome-project-for-early-psychosis/document/hcp-ep), but in brief, the patient group included subjects with DSM-5 diagnosis of schizophrenia, schizophreniform, schizoaffective, psychosis not otherwise specified, delusional disorder, or brief psychotic disorder within 5 years of initial diagnosis. Subjects with affective psychosis were excluded from this study. HCs had no personal and family history of psychosis/schizophrenia, and subjects with a current, treated anxiety disorder were additionally excluded. From the subjects with usable data, all HCs and all patients with a stable schizophrenia diagnosis at 6 months of care were selected for analysis. Data were acquired on a 3-T MRI (Siemens MAGNETOM Prisma). T1w data were collected using an MPRAGE sequence at 0.8 mm isotropic resolution, echo time = 2.22 ms, repetition time = 2.4 s, field of view = 256 mm, and number of slices = 208. dMRI was acquired with an EPI sequence at 1.5 mm isotropic resolution, echo time = 89.2 ms, repetition time = 3.23 s, field of view = 210 mm, number of slices = 92, MB acceleration factor = 2, and flip angle = 78. Ninety-two directions were acquired in both the AP and PA directions at *b* = 1500 and *b* = 3000, along with 7 *b* = 0 images.

### Preprocessing

#### Anatomical Data.

**Fig. 1.**
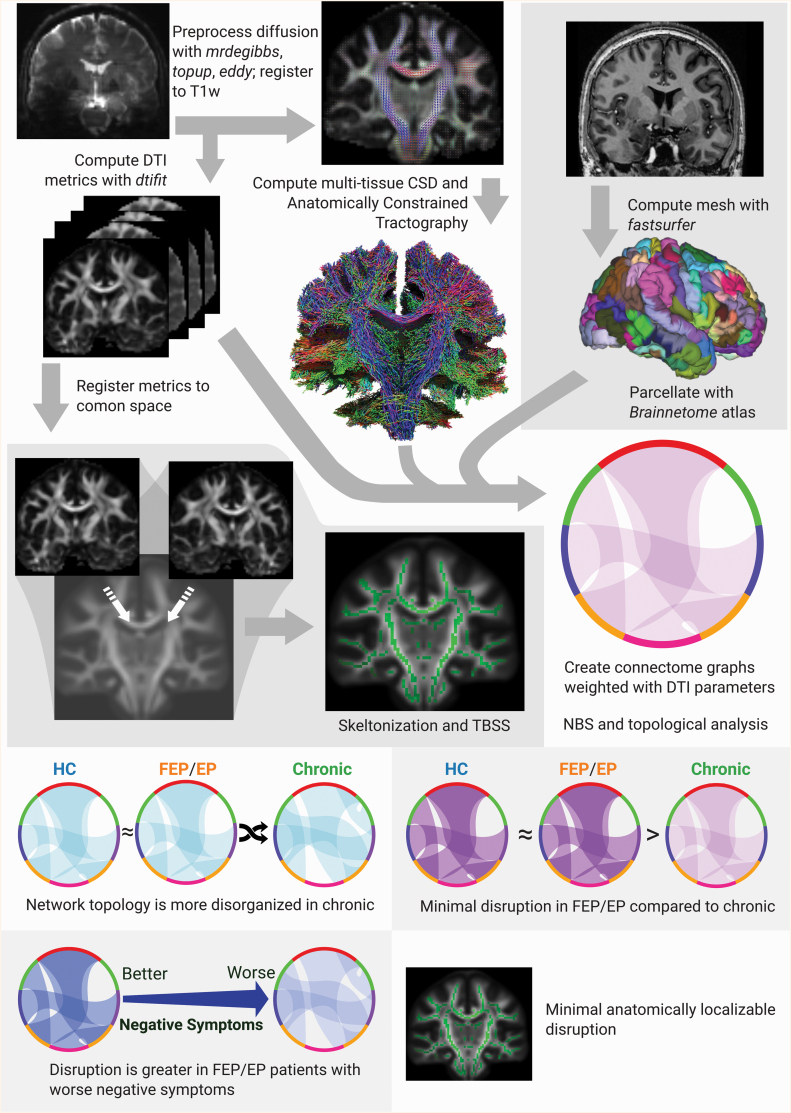
Overview of methodology and results. HC, healthy controls; FEP, first-episode psychosis (in TOPSY dataset); EP, early psychosis (in Human Connectome Project dataset); CSD, constrained spherical deconvolution; DTI, diffusion tensor imaging; TBSS, tract-based spatial statistics; NBS, network-based statistic.

The data analysis pipeline is summarized in figure 1. For TOPSY data, segmentation of the anatomical images and construction of the cortical surface mesh was performed using *FastSurfer*,^57–59^ a recently developed implementation of the cortical parcellation and mesh creation algorithms pioneered by *FreeSurfer*, chosen for its improved processing efficiency and more accurate parcellations (as determined with visual quality control). Remaining processing of anatomical images was done using *ciftify*,^60^ an implementation of the HCP-EP minimal preprocessing workflow.^61^ Of note, images were registered to the *MNI152NLin6Asym*^62^ template space, and meshes were registered to the *fsLR-32k* template space.^63^

For the HCP-EP dataset, we used the minimally preprocessed anatomical images included in the dataset.^61^

#### Diffusion Data.

Diffusion data were preprocessed using *snakedwi*,^64^ a preprocessing pipeline based on *snakebids*^65^ and *snakemake*.^66^ Briefly, Gibbs ringing artifacts were removed with *mrdegibbs* from *MRtrix3*^67,68^; eddy currents and motion were corrected using *eddy* from *FSL*^69^; susceptibility-induced distortions were corrected using *topup* in *FSL*, using the AP-PA pairs of images.^70,71^ The T1w image was skull stripped with *SynthStrip*^72^; bias field correction was applied with *N4ITK* from *ANTS*.^73^ A T1 proxy image was created from the diffusion image using *SynthSR*^74^ and used to accurately register the diffusion image space to the T1w space using a rigid transform calculated with *greedy*.^75^ Diffusion tensor imaging (DTI) metrics were calculated using *dtifit* from *FSL* with linear regression.^76^

Tractography was performed using the *MRtrix3*^68^ software suite. Constrained Spherical Deconvolution (CSD) was performed using the dhollander algorithm to estimate the response functions for WM, gray matter (GM), and cerebrospinal fluid (CSF).^77,78^ Single shell 3 tissue CSD (SS3T-CSD), as implemented in *MRtrix3Tissue* (https://3tissue.github.io/), was performed to obtain WM-like fiber orientation distributions (FODs) as well as GM-like and CSF-like compartments in all voxels.^79^ *mtnormalise* was used to correct for residual intensity inhomogeneities.^80,81^ Each FOD then models the directionally specific diffusion signal attributable to WM within the voxel, increasing the robustness of future processing to crossing tracts.^82^ Tractography was performed using the iFOD2 algorithm^83^ and anatomically constrained tractography (ACT),^84^ with 10 000 000 streamlines,^85^ an FOD amplitude cutoff of 0.06, a minimum streamline length of 4 mm, and a maximum streamline length of 250 mm. The anatomical segmentation used for ACT was obtained using *SynthSeg*.^86^ *Spherical-deconvolution informed filtering of tractograms 2* (SIFT2) was used to correct the streamline counts based on the underlying FOD magnitude.^87^

The HCP-EP dataset was preprocessed in the same manner, except that multi-tissue CSD^88^ was used instead of SS3T-CSD. These different tools were chosen so that the same multi-tissue paradigm could be applied to both datasets. As TOPSY data had only a single shell, the regular multi-tissue CSD algorithm could not be used. All *b*-values were used for the calculation of DTI metrics.

Subject anatomical scans were parcellated using the Brainnetome atlas.^89^ These parcellations were used to derive weighted connectivity matrices based on the tractography data. Average FA, mean diffusivity (MD), radial diffusivity (RD), axial diffusivity (AD), and the log-transformed SIFT2-weighted streamline count were used as weights.

### Analysis

Global averages of FA, MD, RD, and AD were obtained using voxel-based and connection-based approaches. For the voxel-based, a mask was defined using the WM segmentation from *SynthSeg*.^86^ For the connection-based approach, the global average degree of the FA, MD, RD, and AD-weighted connectomes was used. Global averages were compared using 1-way ANOVA with Tukey HSD for the TOPSY dataset, and 2-sample *T*-test for the HCP-EP dataset. Two-tailed comparisons were performed in all cases.

Voxelwise differences in FA, MD, RD, and AD were calculated using tract-based spatial statistics (TBSS).^90^ DTI metrics were used because more complicated models could not be fit to our TOPSY dataset, which has a maximum *b*-value of 1000. FA maps were first nonlinearly registered to a common template corresponding to the average space of all FA images. This template was computed with an in-house implementation of the iterative algorithm described by Avants et al,^91^ using *greedy*^75^ to compute each transformation. The other DTI maps were all transformed into this space. *FSL*^71^ was then used to skeletonize the maps, and *FSL randomise*^92^ with threshold-free cluster enhancement-correction and 10 000 permutations were used to compare groups, thresholding at a corrected *P*-value of .05. Sex and age were regressed as nuisance variables. For HCP-EP data, acquisition site was additionally regressed.

Connectivity differences were assessed using the subject-specific connectomes. Edges with significantly differing weights across groups were identified using the network-based statistic (NBS)^93^ with extent-based cluster sizes, a *T* threshold of 3.0, 10 000 iterations, and corrected *P*-value threshold of .05. Sex and age (and site for HCP-EP) were regressed as nuisance variables.

For all group-by-metric comparisons described above, 1-tailed tests were used with the expectation that patients would have lower FA and higher MD, RD, and AD than HCs, as reported in the ENIGMA mega-analysis.^11^ For comparison with clinical scores (PANSS-8 for TOPSY and PANSS-30 for HCP-EP), scores were fit as a linear predictor of diffusivity and evaluated with 1-tailed *t*-tests, with contrasts for a negative correlation between score and FA, and a positive correlation with MD, RD, and AD.

Node hubness was calculated using a composite score^94^ based on 4 graph theory metrics: degree, betweenness, clustering coefficient, and path length, all of which have been previously used to capture hubness.^95^ These parameters were defined according the weighted definitions given by van den Heuvel et al.^95^ These parameters were calculated for each node, and the nodes were rank-ordered and assigned a score based on their rank. The score was defined as the node’s position in the rank-ordered list divided by the total number of nodes. In other words, each node had a score between 0 and 1, with 0 given to the node with the lowest value, and 1 given to the node with the highest. Such a score was calculated independently for each of the 4 metrics, and the 4 scores for each node were averaged to get the overall hubness score. This entire process was repeated independently for each subject, yielding subject-specific hubness scores for all nodes. For group analyses, these hubness scores were averaged across the subjects within each group.

Differences in hub-based architecture were analyzed by comparing the relative rank order of nodes between and within groups. First, Kendall’s tau^96^ was used to compare hubness rankings for all subject pairs. This metric varies between −1 and 1, where 1 means the 2 lists have the same order, 0 means the 2 lists have uncorrelated orders, and −1 means the 2 are reversed relative to each other. We then calculated the average within-group and between-group Kendall’s tau, ie, the average metric for each subject in 1 group compared to each subject within the same group (within-group similarity), and the average metric for each subject in 1 group compared to each subjects in another group (between-group similarity). Significance was assessed by randomly permuting the groups 10 000 times.

Connection disruption and node hubness were compared based on an analysis described in Klauser et al,^12^ with some modification. To measure the disruption associated with nodes of a given hubness, nodes were selected from the hubness band surrounding threshold *k* + *r* where the kernel radius *r* was set to 0.05. The proportion of disrupted edges connected to these nodes was compared to 10 000 randomly selected groups of nodes of equal size. This analysis was repeated at values 0.1 < *k* < 0.9. This analysis was repeated using the ranked degree of each node, where each node was assigned a value between 0 and 1 based on its position in a degree-ranked list of nodes. Finally, both of these analyses were repeated using a threshold approach. Here, *k* was treated as an upper or lower threshold. For each value of *k*, the subgraph of nodes above or below *k* was considered, and only edges within that subgraph were evaluated. Empirical disruption proportions were compared to 1000 equivalently sized, randomly selected subgraphs.

### Code Availability

The analyses discussed above and resulting figures were made possible by openly available python packages,^97–107^ particularly *graph-tool*,^108^ *pybids*,^109,110^ *nibabel*,^111^ and *nilearn*.^112^ All code used is freely available at https://github.com/pvandyken/paper-CorticalDisruptionFEPMinimal. Links to pipelines used for data preprocessing are listed at that repository.

## Results

### Demographics

In the TOPSY dataset, no significant difference was found between HCs and FEP patients for sex, age, handedness, or SES. A similar lack of significant differences was found between HCs and chronic patients, except that chronic patients were significantly older than the HC cohort (*t*(71) = 3.67, *P* < .001). Both FEP and chronic patients had significantly lower education levels, higher cannabis use, and higher smoking prevalence than controls (table 1). Considered group by group, there were no significant demographic differences between included and excluded subjects, except that excluded FEP patients had significantly fewer smokers than included (χ²(1) = 6.44, *P* = .011).

**Table 1.** TOPSY Demographics

	HC (*n* = 37)	FEP (*n* = 58)	Chronic (*n* = 17)	HC vs FEP	HC vs chronic	FEP vs chronic
Sex (M/F)	24/13	48/10	14/3	χ²(1) = 3.03, *P* = .082	χ²(1) = 0.973, *P* = .32	χ²(1) = 0, *P* = 1
Age	21.70 (3.49)	22.76 (4.62)	28.81 (7.46)	*t*(93) = −1.19, *P* = .24	*t*(51) = −4.76, ***P* < .001**	*t*(72) = −4.02, ***P* < .001**
Handedness (R/L/A)	34/0/3	50/1/7	15/1/1	χ²(2) = 1.06, *p* = .59	χ²(2) = 2.27, *p* = .32	χ²(2) = 1.33, *p* = .51
Education	14.14 (2.18)	12.75 (1.81)	12.81 (2.37)	*t*(91) = 3.32, ***P* = .001**	*t*(50) = 1.97, *P* = .054	*t*(71) = −0.106, *P* = .92
Parental SES	3.08 (1.34)	3.65 (1.35)	3.41 (1.18)	*t*(83) = −1.93, *P* = .057	*t*(51) = −0.865, *P* = .39	*t*(64) = 0.656, *P* = .51
CAST	6.68 (2.77)	12.32 (6.05)	11.93 (7.96)	*t*(85) = −5.28, ***P* < .001**	*t*(50) = −3.56, ***P* < .001**	*t*(63) = 0.201, *P* = .84
AUDIT-C	3.03 (2.19)	2.22 (2.98)	3.33 (2.50)	*t*(80) = 1.36, *P* = .18	*t*(50) = −0.439, *P* = .66	*t*(58) = −1.3, *P* = .2
Smoker (yes/no)	1/36	18/40	8/9	χ²(1) = 9.63, ***P* = .001**	χ²(1) = 13.5, ***P* < .001**	χ²(1) = 0.867, *P* = .35
Cannabis (yes/no)	10/27	35/19	6/9	χ²(1) = 11.1, ***P* < .001**	χ²(1) = 0.344, *P* = .56	χ²(1) = 2.06, *P* = .15
DUP (weeks)—median (IQR)	N/A	21.00 (68.00)	52.00 (89.00)			
DUI (weeks) —median (IQR)	N/A	139.00 (222.50)	104.00 (356.00)			
Exposed to antipsychotics? (yes/no)	N/A	34/24	N/A			
Antipsychotics Day of Scan (Defined Daily Dose)—median (IQR)^a^	0.00 (0.00)	0.25 (0.50)	1.28 (0.99)			
Lifetime Antipsychotics (Defined Daily Dose)—median (IQR)^a^	0.00 (0.00)	0.93 (3.50)	N/A			
SOFAS	82.03 (4.67)	40.86 (12.28)	54.88 (13.89)	*t*(88) = 18.2, ***P* < .001**	*t*(47) = 10.1, ***P* < .001**	*t*(73) = −4.02, ***P* < .001**
PANSS-8 Total	8.00 (0.00)	25.19 (7.03)	14.69 (6.61)	*t*(89) = −14.8, ***P* < .001**	*t*(51) = −6.23, ***P* < .001**	*t*(68) = 5.31, ***P* < .001**
PANSS-8 Positive	3.00 (0.00)	11.98 (2.83)	6.94 (3.60)	*t*(89) = −19.3, ***P* < .001**	*t*(51) = −6.73, ***P* < .001**	*t*(68) = 5.87, ***P* < .001**
PANSS-8 Negative	3.00 (0.00)	7.73 (4.39)	4.19 (1.80)	*t*(90) = −6.53, ***P* < .001**	*t*(51) = −4.07, ***P* < .001**	*t*(69) = 3.13, ***P* = .002**
PANSS-8 General	2.00 (0.00)	5.51 (2.36)	3.62 (2.39)	*t*(90) = −9.01, ***P* < .001**	*t*(51) = −4.19, ***P* < .001**	*t*(69) = 2.8, ***P* = .006**

*Note*: Group distribution columns show *mean* (*sd*) unless otherwise indicated. *P* values less than .05 are shown in bold. M, male, F, female, SES, socioeconomic status, CAST, Cannabis Abuse Screening Test, AUDIT-C, Alcohol Use Disorders Identification Test, DUP, duration of untreated psychosis, DUI, duration of untreated illness, SOFAS, Social Occupational Functioning Assessment Scale, PANSS-8, Positive and Negative Syndrome Scale 8.

^a^Excluding patients with no antipsychotic exposure.

In the HCP-EP dataset, no significant differences between HCs and patients were found for sex, SES. Patients were significantly younger (*t* = 3.51, *P* < .001), more right-handed (χ² = 3.41, *P* < .05), less educated (*t* = 4.95, *P* < .05), and more likely to smoke (χ² = 3.32, *P* < .05) than the HCs. Cannabis use was not reported among HCs, but 29% of patients had at least some exposure. These patients were in treatment for >3 years, with variable medication compliance over this period (table 2).

**Table 2.** HCP-EP Demographics

	HC (*n* = 54)	Patient (*n* = 57)	HC vs Patient
Sex (M/F)	35/19	43/14	χ²(1) = 1.03, *P* = 0.31
Education	15.96 (1.92)	13.29 (1.31)	*t*(39) = 4.95, ***P* < 0.001**
Age	24.89 (4.15)	21.84 (2.82)	*t*(109) = 4.54, ***P* < 0.001**
Handedness (R/L)	43/10	52/3	χ²(1) = 3.41, *P* = 0.065
SES	2.06 (1.05)	2.52 (1.24)	*t*(107) = −2.1, ***P* = 0.038**
Smoke (Yes/No)	3/49	11/46	χ²(1) = 3.32, *P* = 0.068
Pack years	0.02 (0.10)	0.27 (0.71)	*t*(83) = −2.23, ***P* = 0.029**
Cannabis	N/A	1.46 (0.85)	
Antipsychotic duration (months)	0.00 (0.00)	18.05 (15.55)	
Lifetime antipsychotics (Defined Daily Dose)	0.00 (0.00)	234.21 (261.03)	
PANSS30 Total	N/A	51.76 (9.55)	
PANSS30 Positive	N/A	11.58 (3.73)	
PANSS30 Negative	N/A	15.61 (5.25)	
PANSS30 Global	N/A	24.71 (4.51)	

*Note*: Group distribution columns show *mean* (*sd*). *P* values less than .05 are shown in bold. SES, socioeconomic status, PANSS30, Positive and Negative Syndrome Scale.

### Global Changes

We first looked for evidence of global microstructural changes in the WM of FEP and chronic patients. We considered 2 alternative methods to characterize the global WM state. The first is a simple average of all WM voxels. Interpretation of this approach assumes all voxels are of equal import in their interaction with schizophrenia, regardless of anatomical localization. The second approach is a network-based average. For this, we identified cortical and subcortical connections using tractography and the Brainnetome cortical parcellation.^89^ These connections were weighted by the DTI parameters sampled along the constituent streamlines. The weight of each connection was then averaged to get the global average of the network degree. This approach more specifically considers the effect of voxelwise changes on the global network. Voxels with greater connection density, such as those in more central WM regions, are essentially given more weight.

**Fig. 2.**
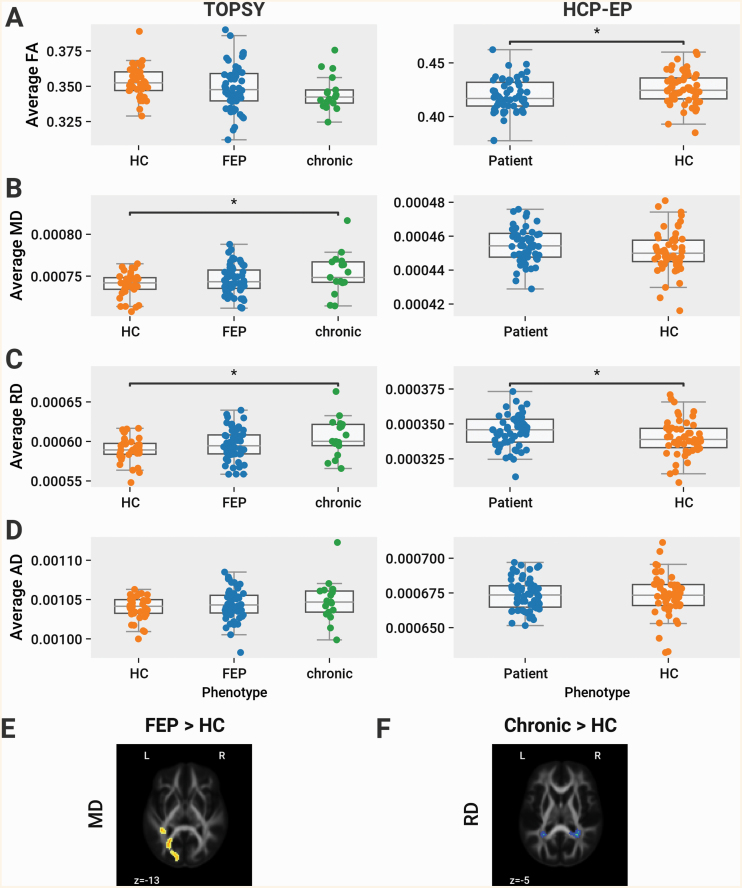
Microstructure across the brain is modestly affected in chronic and early psychosis. (A) Global average of FA across brainnetome connectome connections. Left column shows data from TOPSY dataset, right shows data from HCP-EP. All comparisons within TOPSY are tested using 1-way ANOVA with follow-up post hoc analysis using Tukey’s HSD with the family-wise error rate controlled to 0.05. All comparisons within HCP-EP are tested using 2-sample *t*-test. (A) Average FA. In HCP-EP, a significant decrease of FA was observed in patients (*t*(109) = −2.42, *P* = .017). (B) Average MD. In TOPSY, significant MD differences found between groups (*F*(2,111) = 3.22, *P* = .044), with significantly higher MD in chronic patients compared to HCs (*P* = .036). (C) Average RD. In TOPSY, significant RD differences found between groups (*F*(2,111) = 3.78, *P* = .026), with significantly higher RD in chronic patients compared to HCs (*P* = .02) In HCP-EP, a significant increase was observed in patients (*t*(109) = 2.19, *P* = .031.) (D) Average AD. (E and F) Skeletonized voxels with significantly increased MD and/or AD in HCs compared to compared to (E) FEP and (F) chronic patients, as shown by FSL’s TBSS. Results as shown are inflated for visualization. Region size and extent are shown in supplementary table 2. Results are thresholded for a FWER of 0.05. Abbreviations: MD, mean diffusivity, RD, radial diffusivity, AD, axial diffusivity, TOPSY, treatment outcomes in psychosis, HCP-EP, Human Connectome Project-Early Psychosis.

**Fig. 3.**
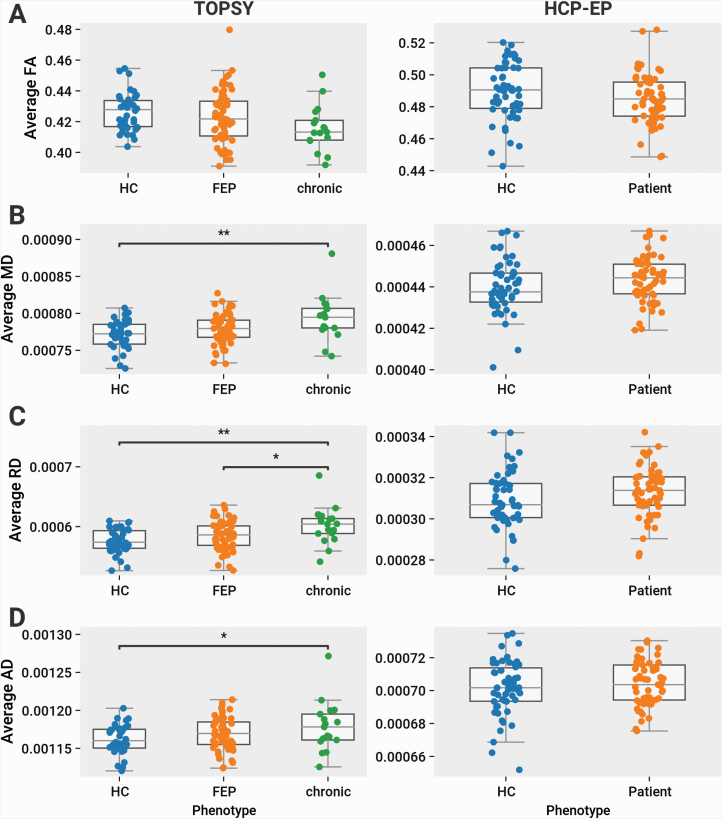
Chronic, but not first-episode psychosis (FEP) and early psychosis (EP) patients, are different from healthy controls (HCs) in network-based averages of DTI parameters. Left column shows data from TOPSY dataset, right shows data from HCP-EP. All comparisons within TOPSY are tested using 1-way ANOVA with follow-up post hoc analysis using Tukey’s HSD with the family-wise error rate controlled to 0.05. All comparisons within HCP-EP are tested using 2-sample *t*-test. No differences were found in HCP-EP in any of the metrics analyzed. (A) Average FA. In TOPSY, no significant differences in FA across groups. (B) Average MD. In TOPSY, significant MD differences found between groups (*F*(2,111) = 6.30, *P* = .002), with significantly higher MD in chronic patients compared to both HCs (*P* = .001) and FEP patients (*P* = .051). (C) Average RD. In TOPSY, significant RD differences found between groups (*F*(2,111) = 6.55, *P* = .002), with significantly higher RD in chronic patients compared to both HCs (*P* = .001) and FEP patients (*P* = .036). (D) Average AD. In TOPSY, significant AD differences found between groups (*F*(2,111) = 3.57, *P* = .032), with significantly higher AD in chronic patients compared to HCs (*P* = .027) but not FEP patients. Abbreviations: MD, mean diffusivity, RD, radial diffusivity, AD, axial diffusivity, TOPSY, treatment outcomes in psychosis, HCP-EP, Human Connectome Project-Early Psychosis.

Considering a simple average of DTI parameters across WM voxels, no differences were found between HCs and FEP patients or between FEP and chronic patients in any of FA, MD, RD, and AD. Between HCs and chronic patients, we observed an increase of MD (*P* = .036) and RD (*P* = .02) (figure 2A–D). With the network-based average degree, between HCs and chronic patients we observed a significant increase in MD (*P* = .001), AD (*P* = .027), and RD (*P* = .001). Compared to FEP, we observed significantly increased RD (*P* = .036) in chronic patients. No differences were found in any other comparison in the TOPSY dataset (figure 3).

In the HCP-EP dataset, using the simple voxelwise average, we observed a significant reduction in FA (*t*(109) = −2.42, *P* = .017) and increase in RD (*t*(109) = 2.19, *P* = .031) in EP patients compared to HCs (figure 2A–D). No changes, however, were found for any parameter using the network-based approach (figure 3).

### Localized Changes

To see if changes could be observed at a regional level, we selected 2 localized approaches corresponding to the voxel-wise and connection-wise approaches described above. TBSS was designed to detect anatomically localized changes in WM tracts and was thus used to explore voxel-based differences. NBS, in contrast, compares network connections^93^ and was used to perform connection-based analysis.

No anatomically localized changes in FA or AD were detected between any group using TBSS, supporting our premise that “lesion-like” localized changes in psychosis are subtle and diffuse. Between HCs and chronic patients, small regions with significant increases in MD (right hemisphere) and RD (bilateral) were found in the posterior junction of the corpus callosum and corona radiata. Between HCs and FEP patients, a region with increased MD was found in the superficial WM of the left occipital lobe (figure 2E and F). The extent and size of affected regions is summarized in supplementary table 2.

No changes were found with TBSS in the HCP-EP dataset.

**Fig. 4.**
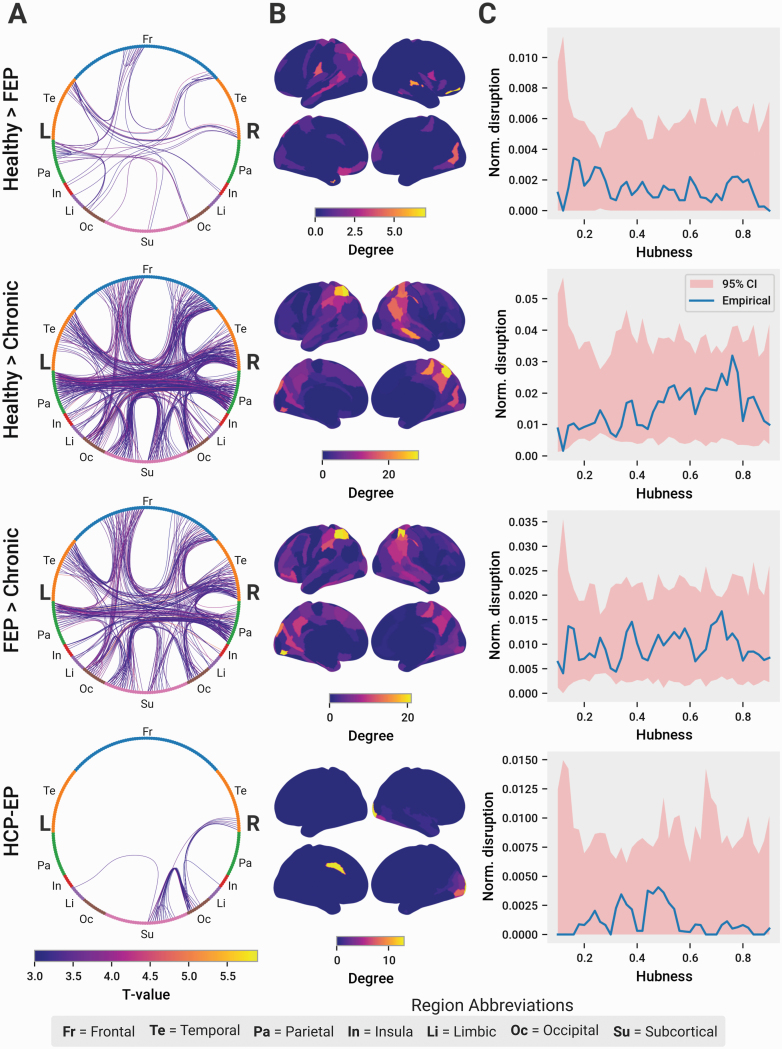
Chronic patients have more disrupted edges than FEP and early psychosis. In all panels, the first 3 rows show data from the TOPSY dataset, the fourth from the HCP-EP dataset. (A) Edges with significantly reduced FA as determined with NBS. Nodes corresponding to the Brainnetome parcellation are represented around the edge of each graph diagram and colored according to lobe they belong to. Left hemisphere nodes are on the left side of the figure, right hemisphere nodes are on the right. Visualized edges are those with significantly lower FA in the control group (FEP in the FEP vs chronic comparison). Edges are colored according the *T*-value determined by NBS. (B) Spatial distribution of nodes with disrupted edges. Regions are colored according to the number of disrupted edges connected to the region. (C) Hubness of disrupted edges. *X*-axis corresponds to a band of hubness values corresponding to the hubness *x* ± 0.05. Nodes within that hubness band are selected and the proportion of disrupted edges connected to those nodes is calculated. This proportion is compared to 1000 randomly selected groups of nodes of equal size. The 95% confidence interval of this random distribution is represented by the red-shaded region.

**Fig. 5.**
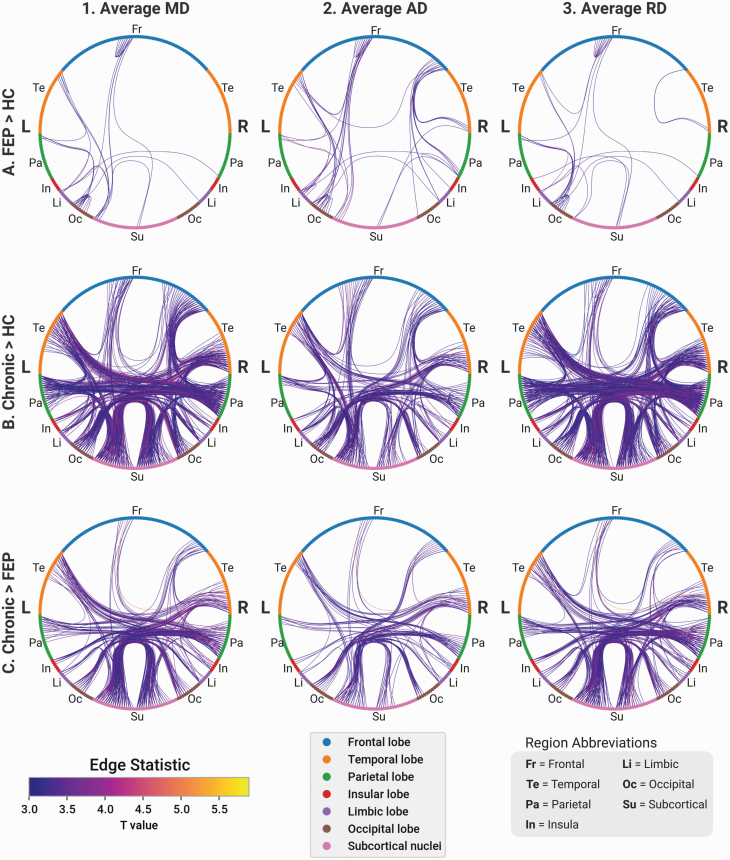
Anatomical connections are altered in FEP and chronic patients. For each network diagram, lines correspond to edges with a significant increase of the corresponding metric in the corresponding group. Nodes are colored according to brain region. Left hemisphere nodes are on the left side of each diagram, right hemisphere on the right. Rows: (A) Edges of chronic patients with increased parameter values compared to HC. (B) Chronic compared to FEP. (C) FEP compared to HC. Columns: (1) Increased average mean diffusivity. (2) Increased average axial diffusivity. (3) Increased average radial diffusivity.

NBS was calculated on the Brainnetome connectomes analyzed above. Between HC and FEP patients, we found subgraphs of around 50 edges with significantly decreased FA (figure 4A) and increased MD, AD, and RD, predominated by intrahemipheric connections (figure 5). Between HCs and chronic patients, subgraphs ranging from 300 to 800 edges were found using the same comparisons. Subgraphs of 150–400 edges were found comparing FEP to chronic patients. Compared to both HCs and FEP, disrupted edges in chronic patients were predominantly interhemispheric, indicating a relative expansion of network-level changes in established cases (figure 5B and C). The edges primarily originated from the temporal cortex, insula, and frontal cortex (figure 4B). In the HCP-EP dataset, a small subnetwork of disrupted connections predominantly in the right occipital cortex was found in EP patients compared to HCs (figure 4A). No changes were found for any other parameter.

**Fig. 6.**
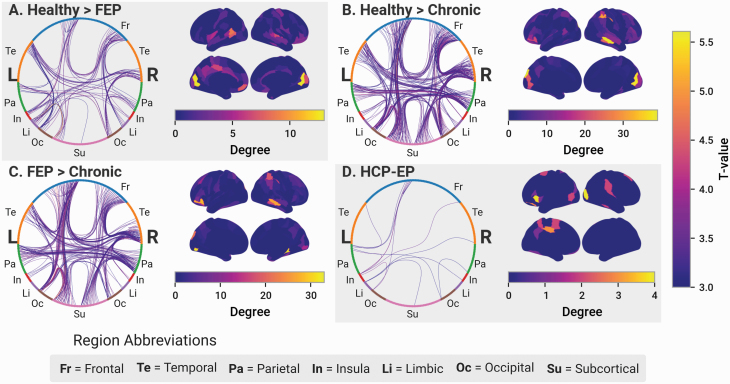
Anatomical connections in schizophrenia patients have reduced streamline count. In the left of each panel, edges with significant effects as determined with NBS represented connecting nodes (*T*_*threshold*_ = 3, *P* < .05). Edges were weighted with the logarithm of the SIFT2-weighted streamline count. Nodes corresponding to the Brainnetome parcellation are represented around the edge of each graph diagram, colored according to lobe they belong to. Left hemisphere nodes are on the left side of the figure, right hemisphere nodes are on the right. In the right, the number of significant connections of each region represented with color. (A–C) TOPSY dataset: (A) Reduced streamline counts in FEP patients compared to controls, (B) in chronic patients compared controls, (C) in chronic compared to FEP patients. (D) HCP-EP dataset, reduced streamline counts in EP patients compared to controls.

We additionally performed NBS to compare the SIFT2-corrected streamline count for each connection, a metric sensitive to the axonal density underlying the computed streamlines.^87,113^ In the TOPSY dataset, all 3 groups had differences between each other, but as before, the smallest subgraph was found in the HC versus FEP patient comparison, the next largest between FEP and chronic patients, and the largest between HCs and chronic patients (figure 6A–C). A small subnetwork with reduced streamline count in EP patients compared to controls was found in the HCP-EP dataset (figure 6D). This indicates that the minimal disruption seen in FEP is not an epiphenomenon of methodological differences in quantifying connectivity.

### Topology

**Fig. 7.**
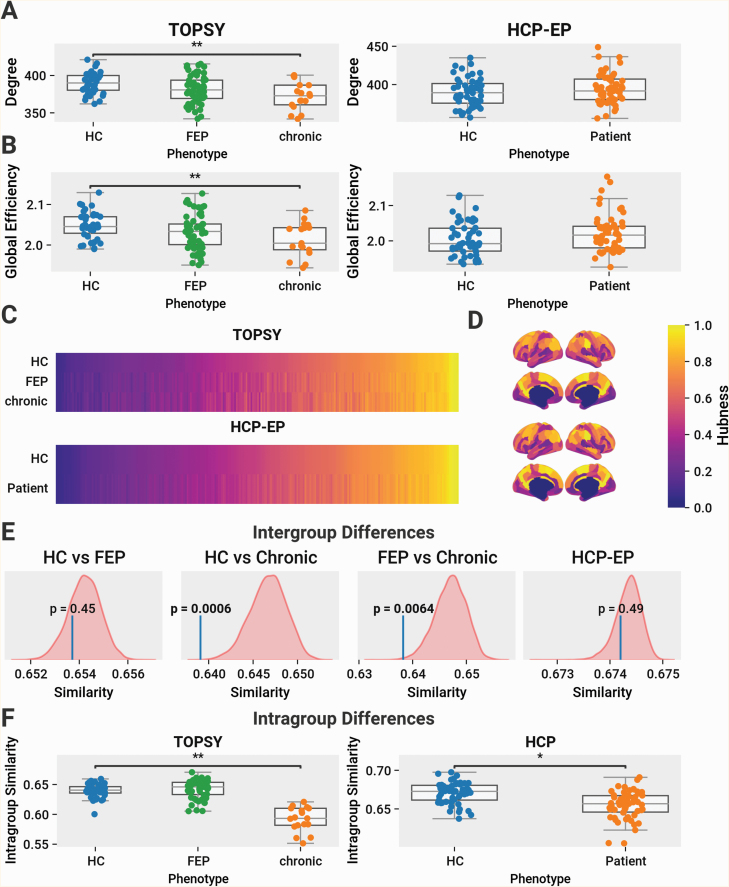
Evidence of topological disruption in chronic but not in first-episode psychosis (FEP) or early psychosis (EP) patients. (A and B) Graph features calculated on the Brainnetome-parcellated connectome weighted with the logarithm of the SIFT2-weighted streamline count. (A) Average degree across all nodes. A significant difference found across groups in the TOPSY dataset (1-way ANOVA, *F*(2,111) = 6.52, *P* = .002), post hoc analysis found a significant reduction in degree compared to HCs in chronic (Tukey HSD, FWER = 0.05, *P* = .002) patients. No differences found in the HCP-EP dataset. (B) Average global efficiency across all nodes. A significant difference found across all groups in the TOPSY dataset (1-way ANOVA, *F*(2,111) = 4.95, *P* = .008), post hoc analysis found a significant reduction in chronic patients compared to HCs (Tukey HSD, FWER = 0.05, *P* = .002). No differences in the HCP-EP dataset. (C) Average rank order of hub nodes across groups. *X*-axis corresponds to the Brainnetome connectome nodes, rank ordered for each graph in order of increasing hubness for the datasets’s HCs. Disease conditions are ordered in the same order as HCs for qualitative comparison. (D) Spatial distribution of nodes. Each region is colored according to its hubness in HCs as shown in C. (E) Permutation analyses comparing similarity of hubness rank-order lists between groups, as calculated with Kendall’s tau. Curve shows the random distribution obtained by 10 000 permutations, randomly shuffling participants between the 2 groups under consideration. The blue line shows the empirical value. Significant *P*-values displayed in bold. (F) Rank-order within-group similarity. Chronic patients have significantly less internal similarity than HCs (permutations = 10 000, *P* = .007) but not FEP patients. The right chart show the same analysis for the HCP-EP dataset. EP patients have less internal similarity than HCs (permutations = 10 000, *P* = .010).

Changes in the cortical hierarchy have previously been associated with schizophrenia. In particular, studies have found disrupted edges to be predominantly between topologically central nodes. To investigate this, we performed a variation of an analysis previously reported by Klauser et al,^12^ which measured the proclivity of connections above a given threshold degree to be disrupted, as determined by NBS. Here, we defined a composite metric of hubness comprising 4 complementary graph theory measures: degree, average shortest path length, betweenness centrality, and the clustering coefficient. All metrics were calculated using the structural connectome weighted with the SIFT2-corrected streamline count. Across all subjects, hubs were situated in locations predicted by existing literature, including the prefrontal cortex, anterior and posterior cingulate cortex, precuneus, and parietal cortices (figure 7C). We measured the probability that edges of a given hubness would be disrupted compared to the general probability of disruption, using a sliding window approach. In all comparisons, there was no particular hubness regime with a disproportionate extent of disruption (figure 4C). This finding remained true when using a threshold version of the approach more comparable to the original paper: instead of nodes within a fixed, sliding kernel, all nodes above a sliding threshold were analyzed (supplementary figure 2A). Finally, no significant results were observed when using an upper-bound threshold instead of a lower-bound (supplementary figure 2B), and when using the rank-ordered degree of nodes instead of hubness (supplementary figure 2C–E), again confirming the robustness of our results to methodological differences.

To investigate changes in the topology and hierarchy of cortical connectivity, we compared the average degree and global efficiency using the SIFT2-weighted structural connectomes. A significant reduction in average degree was observed in chronic (*P* = .002) patients compared to HCs, but not between other patient groups (figure 7A). No changes in global efficiency were found in any comparison (figure 7B). Changes in cortical hierarchy across groups were measured by comparing the rank-ordered list of nodes sorted by increasing within-group average hubness (figure 7C and D) (see supplementary figure 3 for subject-specific hub rankings). Similarity was measured using the Kendall Tau Rank correlation coefficient. The hubness rankings of chronic patients has significantly more within-group variation (*P* = .007) and between-group variation (*P* = .001) than HCs (figure 7E). No differences were found between HCs and FEP patients or between FEP and chronic patients. In the HCP-EP dataset, patients had significantly less within-group (*P* = .010) but not between-group similarity than controls (figure 7F).

Overall these results indicate that topological changes in hub ranks are also more likely in chronic than in untreated FEP subjects. Importantly, the changes observed in chronic patients were also subtle: their hub rankings remain visually similar compared to other subjects (figure 7C). Furthermore, hierarchical disruption in chronic patients cannot be characterized by any stereotyped rearrangements; instead, the individual rankings of patients are increasingly idiosyncratic, contributing to heterogeneity as shown by the decreased within-group similarity compared to HCs (figure 7E).

### Age-matched HCs and Chronic Patients

To see if the differences between HCs and chronic patients were driven by their difference in age, we repeated significant analyses using an age-matched subset of HCs (*n* = 8) and chronic patients (*n* = 13) (using the same age-cutoff for each group). Demographic comparisons between these 2 groups are shown in supplementary table 1. The network degree difference in FA was no longer significant, but chronic patients still had a significantly lower average SIFT2-weighted streamline count (*t*(20) = −2.58, *P* = .018) (supplementary figure 1A). Many connections had significantly lower FA as determined by NBS, although not as many as the main comparison (family-wise error rate = 0.05) (supplementary figure 1B and C). No significant regions were found in the TBSS subgroup analysis.

### Duration of Untreated Psychosis

To see if global FA was related to the duration of untreated psychosis (DUP) in FEP, we modified our TBSS and NBS analyses to test for a correlation between FA and DUP in our FEP patients. *N* = 47 patients had a valid DUP that recorded based on reliable historical account of the time of onset of first psychotic symptom. The mean DUP was 60.17 weeks, but the median was 21, reflecting a highly left-skewed distribution. We thus took the log of DUP before applying the regression with FA. No clusters were significantly associated with DUP in our TBSS analysis, and no significantly disrupted subnetwork was found with NBS. This analysis was not repeated in the HCP-EP data, as patients in that dataset have a variable illness duration, obfuscating any potential effect of DUP.

### Clinical Scores

The TBSS and NBS paradigms described above were used to test for correlations between DTI metrics and the positive and negative symptom score (PANSS-8) symptom scores in FEP and PANSS-30 in EP patients. No global correlations were found between the subscores and the PANSS subscsores, and no differences were found with TBSS. Subgraphs with significant correlations were, however, found using NBS, particularly a negative correlation between FA and the negative subscore in both the TOPSY and HCP-EP datasets. In the TOPSY FEP patients, smaller subgraphs had a positive correlation between MD, RD, and AD and the PANSS8-P subscore, and in the HCP-EP EP patients, subnetworks were found with positive correlations between these same parameters and PANSS30-N, indicating that DTI disruptions primarily vary with individual differences in the burden of negative rather than positive symptoms.

## Discussion

**Fig. 8.**
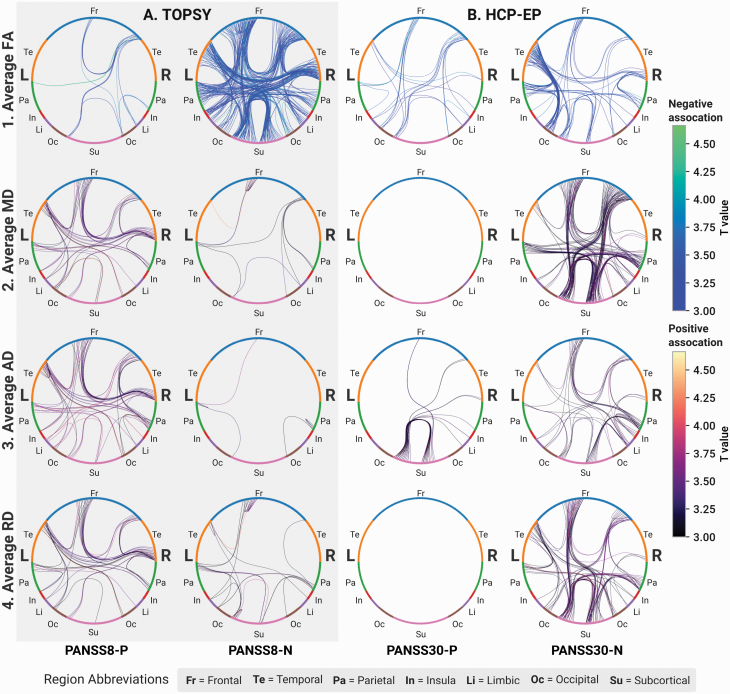
Microstructure of anatomical connections correlates with PANSS subscores in FEP and EP patients. For each network diagram, lines correspond to edges with a significant increase of the corresponding coefficient in the corresponding group. Nodes are colored according to brain region. Left hemisphere nodes are on the left side of each diagram, right hemisphere on the right. In A and B, the left column shows correlations with the positive subscore, the right correlations with negative: Columns: (A) FEP patients from TOPSY dataset with PANSS-8 subscores. (B) EP patients from HCP-EP with PANSS-30 subscores. Rows: (1) Decreased average fractional anisotropy. (2) Increased average mean diffusivity. (3) Increased average axial diffusivity. (4) Increased average radial diffusivity.

Focusing on the nature of WM changes in FEP, EP, and chronic schizophrenia, we report 2 key observations: (1) in both untreated FEP and treated early stages of psychosis, structural changes of the WM are minimal (figure 4), tend to vary with the severity of negative symptoms (figure 8), and are restricted to superficial, posterior, and peripheral WM regions; and (2) the topological properties of the anatomical networks of FEP patients remain intact, with the exception of a subtle reduction in the overall degree of connectivity based on SIFT2-weighted streamline count. Contrasted with the evidence for WM disruption in chronic schizophrenia from both the extant literature and our own chronic sample, these results indicate that focal WM changes may not be necessary causal factors for the onset of a psychotic episode but may play a role in progression of the illness to chronic stages.

Given the system-wide patterns of disruption observed in chronic patients^12^ and the long functional decline that often precedes the first psychotic episode,^24^ 1 might expect a widespread, accumulated disruption of connectivity in FEP patients. Observation of such disruption would strengthen the case for the primacy of structural dysconnectivity in the clinical expression of psychosis. However, our results demonstrate that connectivity deviations at the resolution measurable by MRI are minimal by the time of the first psychotic episode, restricted to those who have more prominent negative symptoms, eliminating extensive WM structural decline as a necessary precondition for the expression of positive psychotic symptoms. The pronounced WM disruption seen in established cases of schizophrenia likely reflects secondary mechanisms distinct from the causal pathway of positive symptom expression.

Some limited evidences for early microstructural changes were observed in our data, including the increased localized AD observed in FEP patients with TBSS (figure 2E), and the global changes in FA and RD in the HCP-EP dataset (figure 2A and C). Given the other negative results, however, these seem to represent more diffuse or peripheral changes than would be found in chronic counterparts. In the TOPSY dataset, the increased MD was centered in the superficial WM of the occipital lobe, away from central callosal or association tracts. This corresponds with the paucity of disrupted connections as determined with NBS. Since central WM regions have a higher connection density, centralized disruption would result in greater connection disruption. Similarly, although the HCP-EP dataset shows global changes, they are not sufficiently concentrated in any 1 region to manifest either in TBSS or NBS. Although we were not able to localize these changes, we note their consistency with peripheralized change, as TBSS can fail to properly align voxels in more peripheral WM regions, and NBS is inherently less sensitive to peripheral change due to the lower connection density.

In contrast to the DTI results, reductions in SIFT2-weighted streamline count were observed both in FEP, EP, and chronic patients, albeit with a greater extent in the chronic schizophrenia group. The SIFT2 weighting is derived from the WM FODs along the length of the tract, which in turn model the magnitude and direction of the diffusion signal attributable to the intra-axonal tissue compartment. A reduced weight thus reflects a reduced magnitude of this compartment, which could, eg, result from a reduction in fiber density or diameter.^87^ Compared to the postmortem studies of neuronal soma, axonal properties are less well studied in schizophrenia, though altered axonal morphology has been reported in relation to longer illness duration and more prominent negative symptoms.^114^ While the reduced streamline count in chronic compared to FEP patients supports our overall conclusion that FEP patients have reduced disruption of connectivity compared to chronic, the appreciable decline of degree in FEP patients compared to HCs suggests this metric may be a more sensitive marker of EP than traditional DTI metrics. Importantly, however, only a very small significant subgraph was found in the HCP-EP dataset, and its multiple b-values allow a more specific reconstruction of WM diffusion signal compared to our TOPSY data. More work will thus be needed to clarify the generalizability of this parameter.

Despite changes in streamline count, FEP patients maintain the structural hierarchy of the WM network. Across all participants in both datasets, the hub nodes are generally focused in the prefrontal cortex, cingulate gyrus, and precuneus (figure 7E), with high quantitative similarity between individuals (figure 7F). Chronic patients alone have a subtle, yet significant decrease in hub rank similarity compared to HCs, both within-group and between groups, suggesting more idiosyncratic connectome organization in later illness stages (supplementary figure 3E). Chronic patients also show a decrease in global network efficiency not observed in FEP patients.

Finally, although FEP and EP patients as a bloc minimally differ from HCs, we did find linear correlations between diffusion parameters and the PANSS symptom scores, particularly the negative syndrome score, in both datasets. This is consistent with Kochunov et al^48^ who observed a specific association between WM (but not GM) changes and negative (but not positive) symptoms, despite finding minimal WM changes in FEP (defined as <5 years of illness). Negative symptoms are a pervasive feature of schizophrenia,^115–117^ with a higher symptom burden linked to poorer hospitalization outcomes^118^ and degraded social and occupational functioning.^24^ Since our results suggest there remains interplay between structural connectivity and determinants of long-term outcomes in schizophrenia, the possibility remains of using these imaging metrics for risk stratification.

### Potential Sources of Change

Both anatomically and topologically, we observe disparities between FEP and chronic patients. Three potential noncausal mechanisms may contribute to this.

First, structural decline may be a secondary phenotype of upstream disease processes. Although we lack longitudinal data to conclusively demonstrate this hypothesis, we cite 3 lines of evidence supporting this notion. First, functional connectivity is known to be disrupted in FEP,^119–121^ and long-term interregional signaling patterns modify the strength of the connecting tracts,^122,123^ and can affect tract myelination.^124^ Thus, aberrant functional signaling, over time, may lead to reorganization of the anatomical network. Second, schizophrenia has been associated with neuroinflammation and myelin degradation. For instance, a meta-analysis by Najjar et al found strong evidence for neuroinflammatory pathology in the WM of chronic schizophrenia patients.^125^ Third, several studies of FEP have found aggravated findings resulting from a longer DUP. Kraguljac et al found patients with longer DUP had lower global FA levels.^27^ A more focused study of the tapetum found a similar result.^126^ Note that Filippi et al have reported the opposite trend.^33^ In our own results, we did not observe a relationship between DUP and FA, however, our participants were significantly skewed toward low DUP, limiting our ability to observe an effect. More study of the progression of untreated psychosis is needed to understand the impact of early intervention and the disease-halting potential of medication.

A second source of change may be the psychoactive drugs used therapeutically by schizophrenia patients. Since most participants with chronic schizophrenia are undergoing treatment and treatment cannot be ethically withheld, disambiguating the effects of the disease and treatment is generally an ill-posed problem. A few studies, however, have achieved natural cross-sectional experiments. In a rare sample of 17 unmedicated patients with chronic schizophrenia and an age and illness-duration-matched group of treated patients, Luo et al compared WM integrity^127^ between groups. Unmedicated patients had slightly greater deviation from controls than treated patients, indicating that antipsychotics may not be contributing to the reduced WM integrity per se. Another report found no difference in FA reduction between medicated and unmedicated patients.^12^ Thus, evidence to date is more suggestive of antipsychotics either ameliorating or limiting the WM deficits associated with psychosis, rather than contributing to them.

Finally, network disruption may not result from schizophrenia or its treatment at all. Instead, a subset of early-stage patients with lower WM integrity may preferentially progress toward chronic stages of illness. If so, this would introduce a selection bias when recruiting a sample of established cases of schizophrenia. The enrolled patients are likely to be the ones with severe-enough illness that prompted continued engagement with the health care system. For the TOPSY dataset, we recruited from a consecutive sample of all referrals to the only first-episode program within our catchment, including patients irrespective of later severity and retention probability. Thus, our FEP cohort may display a broader array of neurobiological phenotypes, which, on average, becomes indistinguishable from HCs. This would create an opportunity to find subgroups of patients with greater deviation, which might in turn be predictive of long-term outcomes. Such exploration will be a focus of future work.

### Comparison to Other Works

Our results stand in contrast to some reports. In chronic schizophrenia, most disrupted structural connections have been observed between the highly connected hub nodes found in the rich club of the brain.^12,95,128^ We did not observe this pattern of disruption between any of our groups. This may be due to our relatively small sample size of chronic patients. A study by Cui et al^44^ reports a reduction in connectivity among the rich-club nodes in FEP, and another found generalized disruption of network efficiency and reported fewer hubs in FEP,^45^ but we were not able to replicate this in our datasets. To be sure, our FEP and EP datasets are sufficiently powered and larger than many prior studies that report WM deficits in patient groups. While this may be due to a greater patient heterogeneity discussed above or a study effect stifling “negative” findings such as we report here, we cannot exclude the potential effects of DUP,^27,126^ age, scan parameters, and processing choices in these differences. We note, however, that unlike data used in previous studies, the HCP-EP dataset is freely available for research allowing for future replicability.

### Limitations

The HCs recruited for our analysis were age-matched to our FEP patients, making age a pertinent difference between both of these groups and our chronic patients. Age has been previously associated with FA decline, but the onset of this decline is typically observed between 40 and 50 years of age.^46,129–131^ Our chronic patients have a mean age of 30, too early for age-related changes to have effect. Accordingly, our post hoc analysis between chronic patients and an age-matched subgroup of controls still found significant FA reductions in the patients, and NBS found a large number of disrupted edges. Although elimination of an age effect could partially explain the reduced effect size, this cannot be distinguished from the loss of power resulting from the smaller sample.^30^

The TOPSY study recruited more FEP than chronic patients, affecting the validity of direct comparison between these groups. We thus consider the lack of difference between FEP patients and HCs to be the more salient result. Differences between HCs and chronic patients are reliably reproducible in the literature; our own reproduction, including in an age-matched subset of HCs, reassures that our dataset bears this basic feature.

### Conclusion

Our results indicate that an extensive pre-onset disruption of WM tracts contributing to the development of psychosis is less likely, though spatially limited changes affecting streamline counts and the topology (degree) are already apparent in untreated FEP. These localized changes in FEP are restricted to the periphery and fail to impact global connectivity in the manner observed in chronic patients. When considered with the accumulating evidence discounting prominent GM changes preceding FEP,^132^ these findings suggest reduced WM integrity in schizophrenia may reflect an accumulated burden wrought by severe mental illness over a sufficiently long period of time, rather than an upstream cause of psychotic phenotypes.

## Supplementary Material

sgae010_suppl_Supplementary_Materials
