# Supplementary material for: Cortical Network Disruption Is Minimal in Early Stages of Psychosis
Source: Schizophr Bull Open. 2024 Apr 22;5(1):sgae010. doi: 10.1093/schizbullopen/sgae010 (PMC11207789; doi:10.1093/schizbullopen/sgae010)
Supplement: sgae010_suppl_Supplementary_Materials [file sgae010_suppl_Supplementary_Materials.pdf]

# 1 SUPPLEMENTARY FIGURES

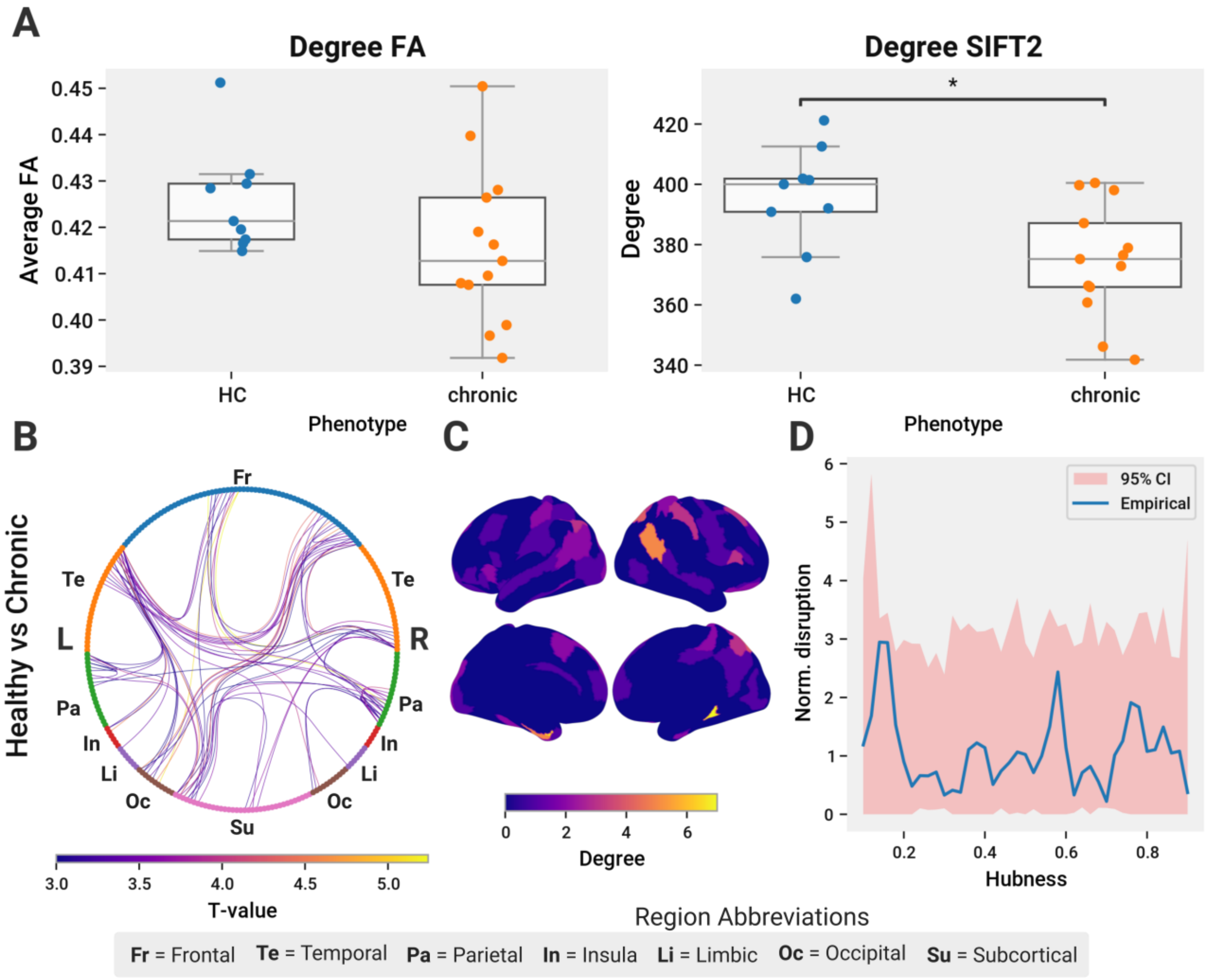


*Figure S1: Age-matched comparisons between &HCs and chronic patients. A. Left: Average FA across all connections. Right: average degree of the Brainnetome-parcellated connectome weighted with the logarithm of the SIFT2-weighted streamline count. Significant reduction in chronics compared to &HCs ($t(20) = -2.58, p = .018$). B. Edges with significantly lower FA in chronic patients compared to age-matched controls. Nodes from the Brainnetome parcellation are colored according to their brain region (as in Figure 7). Edges are coloured according to their T-value. C. Cortical regions colored according to the number of disrupted edges connected. D. Topological organization of disrupted edges, as described in (Figure 7).*

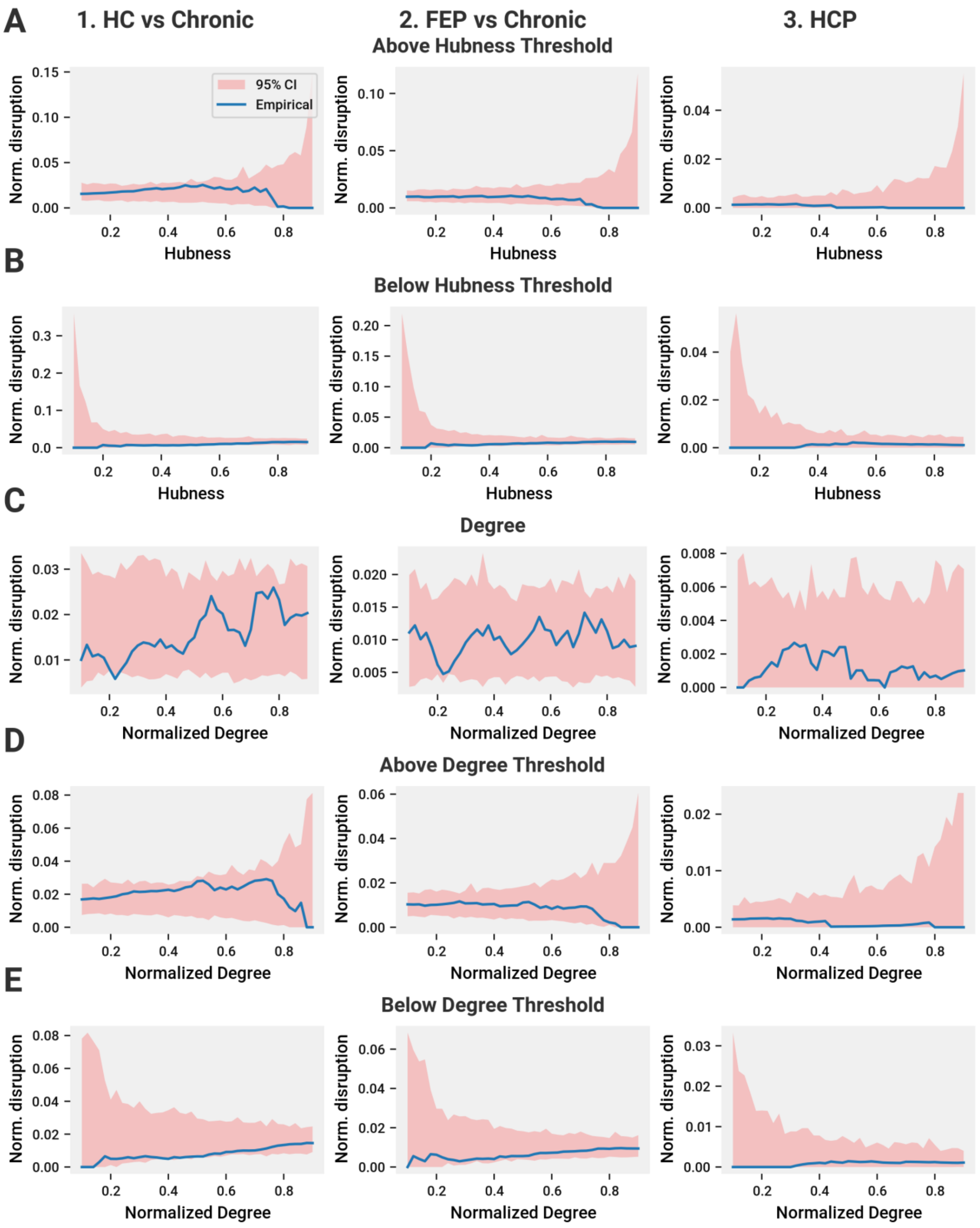


*Figure S2: Alternative measurements of disruption topology fail to find patterns. Charts represent the predominance of edges with significantly reduced FA in various subgraphs. In all rows, the first two columns show data from the TOPSY dataset, the third from the HCP-EP*

dataset. The red shaded regions represents the 95% confidence interval of 10,000 random permutations (re-analyzed at each threshold value). For each permutation, a random subgraph equal to the size of the empirical subgraph of the respective threshold value is analyzed. A. X-axis charts a sliding lower-bound threshold of hubness. At each threshold, the proportion of edges disrupted in the subgraph of nodes with hubness higher than the threshold is graphed on the y axis. B. As in A, but the X-axis represents a sliding upper-bound threshold. C. As in (Figure 7), the X-axis represents a sliding window. For each hubness value $x$, the proportion of edges with significantly lower FA from the entire graph that connect to nodes with hubness equal to $x \pm 0.05$ is calculated. Degree rank is used as the hubness measurement instead of the composite hubness score. D. As in A, but with degree rank. E. As in B, but with degree rank.

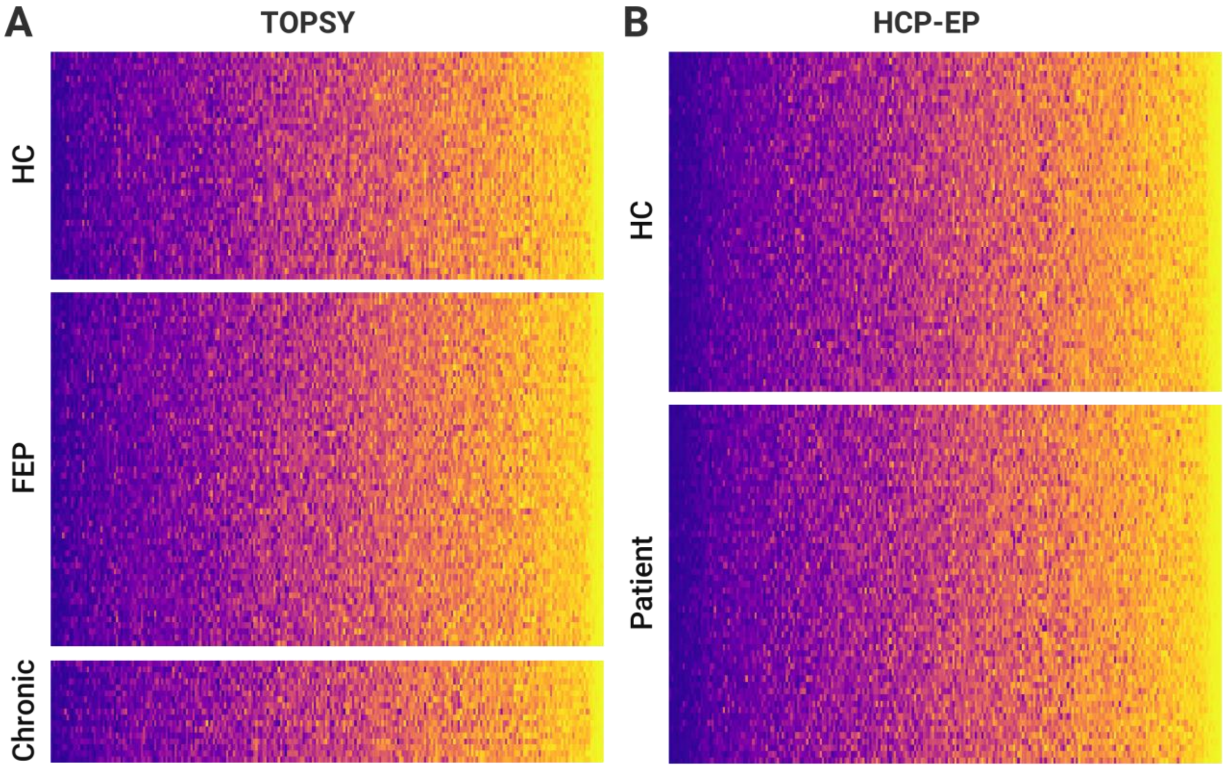


Figure S3: Extended data showing comparisons of node hierarchy. Node hubness rankings for individual subjects. Nodes are rank-ordered along the x-axis according to increasing average hubness across all subjects. The left column shows TOPSY data, the right shows HCP-EP data. Each diagram is split into sections corresponding to the different patient subgroups. The relative ordering of nodes along the x-axis differs between the two datasets, but remains consistent within each dataset across all groups.

# 2 SUPPLEMENTARY TABLES

***Table S1:*** *Demographics of healthy control cohort age-matched to chronic patients.*

| | HC (n=9) | chronic (n=13) | HC vs chronic |
|---|---|---|---|
| Sex (M/F) | 7/2 | 11/2 | $\chi^2(1) = 0$, $p = 1$ |
| Age | 26.44 (1.94) | 30.54 (7.23) | $t(20) = -1.65$, $p = .12$ |
| Handedness (R/L) | 9/0 | 12/1 | $\chi^2(1) = 0$, $p = 1$ |
| Education | 15.33 (2.06) | 12.69 (2.46) | $t(20) = 2.64$, $\mathbf{p = .016}$ |
| SES | 3.89 (1.05) | 3.54 (1.20) | $t(20) = 0.707$, $p = .49$ |
| CAST | 6.44 (1.33) | 10.25 (6.69) | $t(19) = -1.67$, $p = .11$ |
| AUDIT-C | 3.33 (1.80) | 3.25 (2.67) | $t(19) = 0.0807$, $p = .94$ |
| Antipsychotics Day of Scan (Defined Daily Dose) - median (IQR) | 0.00 (0.00) | 1.16 (0.95) | |
| Smoker (yes/no) | 0/9 | 6/7 | $\chi^2(1) = 3.62$, $p = .057$ |
| Cannabis (yes/no) | 5/4 | 4/8 | $\chi^2(1) = 0.328$, $p = .57$ |
| SOFAS | 82.17 (2.32) | 55.46 (13.61) | $t(17) = 4.71$, $\mathbf{p < .001}$ |
| PANSS-8 Total | 8.00 (0.00) | 15.31 (6.80) | $t(20) = -3.2$, $\mathbf{p = .004}$ |
| PANSS-8 Positive | 3.00 (0.00) | 7.38 (3.62) | $t(20) = -3.61$, $\mathbf{p = .001}$ |
| PANSS-8 Negative | 3.00 (0.00) | 4.00 (1.63) | $t(20) = -1.82$, $p = .083$ |
| PANSS-8 General | 2.00 (0.00) | 4.00 (2.52) | $t(20) = -2.37$, $\mathbf{p = .028}$ |

***Table S2:*** *Regions characterized as significantly changed with tract-based spatial statistics.*

| Comparison | Parameter | Num | Extent | Volume ($mm^3$) |
|---|---|---|---|---|
| Chronic > HC | RD | 47 | 0.34% | 376 |
| FEP > HC | MD | 142 | 1.02% | 1136 |
